# Perspectives towards cultural competence and receptivity to cultural competence training: a qualitative study on healthcare professionals

**DOI:** 10.1017/S1463423623000245

**Published:** 2023-05-26

**Authors:** Daniel W. L. Lai, Vincent W. P. Lee, Yong Xin Ruan

**Affiliations:** 1 Faculty of Social Sciences, Hong Kong Baptist University, Hong Kong, China; 2 Department of Social Work, Hong Kong Baptist University, Hong Kong, China; 3 Department of Social Work, The Chinese University of Hong Kong, Hong Kong, China

**Keywords:** cultural competence training, ethnoculturally diverse patients, healthcare professionals, Hong Kong, professional development

## Abstract

**Background:**

Cultural competence training plays an effective role in improving cultural competence for healthcare professionals, but cultural competence training was found to be insufficient in Hong Kong.

**Aim:**

This study aims to explore receptivity and readiness of Hong Kong healthcare professionals (nurses, occupational therapists (OTs), and physiotherapists (PTs)) towards cultural competence training.

**Methods:**

Twenty-three semi-structured interviews were conducted with 7 educators/trainers from tertiary institutions, 2 representatives of professional groups, and 14 managerial and frontline workers. Data were analysed using theoretical thematic analysis.

**Findings:**

Results show that nurses and PTs have lower levels of cultural competence than OTs owing to insufficient in-depth training and the nature of professional practice, and they expressed lower willingness to receive the training than OTs. However, the staff in these three professions encounter various challenges in serving ethnoculturally diverse groups. Therefore, barriers in receiving cultural competence training and best practice for providing cultural competence training were identified and discussed for these three professions.

## Introduction and background

Hong Kong is a multicultural society, with an increasing number of non-Chinese residents. The ethnoculturally diverse population in Hong Kong, including people with Filipino, Indonesian, and other Asian backgrounds, increased by 41% to 263 593 between 2006 and 2016 (Census and Statistics Department, 2016). Ethnoculturally diverse groups often encounter challenges particularly in healthcare (O’Connor, 2010). They face difficulty accessing healthcare services owing to language barriers and cultural differences (Lee, 2013; Kapai, 2015), discrimination, and lack of cultural understanding by healthcare professionals (Vandan et al., 2019). They experience health inequalities, such as lower uptake of health screening and poorer health outcomes, compared to Chinese patients (Chen and Chan, 2014; So et al., 2017). This highlights the importance of culturally competent services for ethnoculturally diverse groups. However, healthcare professionals in Hong Kong report a limited ability to provide culturally competent care (Vandan et al., 2019). Cultural competence training for them is therefore needed to strengthen service provision. This requires exploring perspectives on cultural competence training among healthcare professionals (nurses, occupational therapists (OTs), and physiotherapists (PTs)), which remain understudied. The reasons for focusing this study on these three professions include: (1) The research team members, due to professional affiliation and personal networks, have a stronger connection with potential key informants working in these three professions. (2) Considering the challenges faced by ethnoculturally diverse population in areas of access and service use in healthcare settings, the daily practices of three professions would probably involve prolonged in-person-interactions with ethnoculturally diverse patients in the community health settings and in medical settings such as clinics and hospitals. The professional practice contexts are generally different from medical doctors in Hong Kong who normally would only have relatively brief encounters with patients during out-patient consultations or appointments.

For the purpose of this study, cultural diversity is the context in which uniqueness and differences of people would create new opportunities and challenges in social and professional interactions. It concerns with not only differences in people’s race and ethnicity but also other associated characteristics such as education and socio-economic backgrounds (Gigerm cited in Cai, 2016: 271). In healthcare settings, culture is believed to be a factor leading to patients’ expressions of problems, help-seeking behaviours, and responses to treatment (Kirmayer, 2012). In social settings, a culturally diverse society would facilitate mutual awareness, respects, trust, and development of synergy among people from unique background (Josefová, 2014). Cultural diversity is believed to be the antecedent of cultural competence (Cai, 2016). Thus, with the increased ethno-cultural diversity experienced due to immigration and globalization in the western societies (Furness, as cited in Danso, 2018), cultural competence has emerged as a widely recognized, powerful practice to address needs of people from culturally diverse backgrounds (Kirmayer, 2012; Di Stefano et al., 2017; Danso, 2018). It enables healthcare professionals to facilitate accessibility and enhance quality of medical services for ethnoculturally diverse groups (Kaur, 2016). However, Hong Kong as a predominantly Chinese society in the East, cultural diversity is relatively a new phenomenon faced by service providers and healthcare professionals.

‘Cultural competence’ refers to an attitudinal and ethical sense that individuals and institutions are capable to respond respectfully and effectively to people of all cultures, languages, ethnicity, faiths, and other attributes (Lum, 2011). Cultural competence in healthcare refers to the ability to acknowledge, appreciate, and respect clients’ values, preferences, and expressed needs and ‘to resolve differences and identify solutions that reduce interference from various cultural factors’ (Leininger and MacFarland, cited in Lin et al., 2017: 174). It is a level of performance and ongoing joint efforts of healthcare system, organizations, and professionals to provide high-quality care for patients (National Quality Forum, 2008; Cai, 2016). Therefore, the practice of a professional could not be simply judged as culturally competent/incompetent. Cultural competence should be a goal that healthcare professionals and systems/organizations continuously strive to achieve (Castillo and Guo, 2011). Furthermore, cultural and linguistic differences between healthcare providers and health service users can result in significant miscommunication, service user mistrust, decreased satisfaction, and disempowerment (Jongen et al., 2018). In contrast, the practitioners’ increased cultural competence has been linked to increased patient satisfaction, treatment adherence, and information seeking and sharing (Jongen et al., 2018).

Previous research has shown that among nurses, low awareness of patients’ culture and values can cause conflict and ethical dilemmas, negatively influence care outcomes, and fail to adequately address the needs of ethnoculturally diverse groups (Donnelly, 2000; Al-Atiyyat, 2009; Long, 2012). Among OTs and PTs, cultural competence increases consideration of clients’ culture during evaluation and intervention and can support relationships with patients from other cultural backgrounds, leading to productive interventions (Sood and Cepa, 2014; Govender et al., 2017). Therefore, cultural competence has gained increasing attention in healthcare literature (Kirmayer, 2012; Cai, 2016; Di Stefano et al., 2017; Danso, 2018).

To enhance cultural competence of healthcare professionals, some scholars have recommended adding cultural competence components to lectures and classes for healthcare professionals in training (Sargent et al., 2005; Long, 2012; Jacob et al., 2016). Secondly, increasing clinical experience in serving culturally diverse patients can help develop cultural competence, complemented by clinical cases to enrich students’ cultural knowledge (Amerson et al., 2015). Thirdly, an immersion approach (e.g. studying abroad) can be used to enhance cultural competence through cultural competence courses and prerequisite and ongoing learning (Amerson, 2012; Ingulli et al., 2014; Harkess and Kaddoura, 2015). Immersion into environments with different cultures has been shown to support better cultural competence results among nursing students (Amerson et al., 2015). A recent scoping review has noted that there is diversity in methods and strategies for cultural competence and that there is no standardized approach to competence framework development (Batt et al., 2019). It has called for improved guidance in the process for developing and reporting competence frameworks.

In Hong Kong, elements related to cultural understanding appear to have been included in the training curriculums of nurses, OT, and PT. The Nursing Council of Hong Kong’s (2015) *Code of Ethics and Professional Conduct* states that cultural and religious influences on health should be included in nursing training curricula. Additionally, a report by Hospital Authority (2017) in Hong Kong indicates the importance of training in communication with culturally diverse patients and knowledge of multiculturalism for frontline staff. Cultural awareness is listed as a learning outcome for OT programmes and cultural sensitivity is covered in PT training courses (Hong Kong Polytechnic University, 2017; Tung Wah College, 2018). Nevertheless, to facilitate better promotion and implementation of cultural competence, it is worthwhile to examine the perspectives of the healthcare professionals and their views towards training effective to improving cultural competence in practice.

### Analytical framework

In view of the increasingly culturally diversified environment of the healthcare sector in Hong Kong, there is a need to understand the views towards cultural competence training among healthcare professionals and explore how to improve cultural competence training for them. The analytical framework of this study is based upon the Cultural Competence Attainment Model (McPhatter, 1997), which considers how practitioners move towards cultural competence and view it as a long-term, ongoing developmental process. It delineates three essential and interconnected components:

1. **Enlightened consciousness** is about reorienting or restructuring one’s worldview and belief systems and considering aspects of one’s own culture in order to understand other cultures, with attention to influences of socialization, previous environments, and interactions with cultural difference.
2. **Grounded knowledge base** refers to critical analysis of gaps and weaknesses in one’s knowledge and content and biases of previous education, and development of new knowledge (e.g., different cultures, social issues affecting different groups, oppression and discrimination, alternative theoretical and practice perspectives) based on information from diverse communities, institutions, and disciplines.
3. **Cumulative skill proficiency** is a focused, systematic, and reflective process of ongoing development of skills and worldviews, cross-cultural communication, multi-level analysis and intervention, and engagement with culturally diverse clients.

These three components guided the formation of the structure of this study, including previous training, content of cultural competence, approaches to and skills in encountering and accepting difference, and personal and professional values and norms. Based on this analytical framework, the research objectives are enumerated below:

1. Examine understandings and implementation of cultural competence among healthcare professionals, including nurses, PTs, and OTs;
2. Identify facilitators and barriers related to the healthcare professionals’ readiness to receive cultural competence training; and
3. Examine how cultural competence could be more effectively promoted and enhanced among the healthcare professionals.

## Research methods

### Research participants and sampling

In-depth key informant semi-structured interviews for this study were conducted with a range of healthcare stakeholders. Interview participants included healthcare service professionals – registered and enrolled nurses, PTs, and OTs – responsible for providing support to ethnoculturally diverse groups, and other stakeholders influencing support and service provision including educators or trainers from tertiary institutions and trade union representatives. Participants of the interviews were identified using a purposive sampling method. The research team engaged their existing social and professional networks and searched relative agencies online including academics from training institutions and health professionals in managerial and frontline positions. They were recruited as long as they had accredited qualification in their professions. A total of 23 interviews with healthcare professionals were conducted, including 7 educators or trainers from tertiary institutions training healthcare professionals (5 from the nursing field, 2 from the OT/PT field), 2 trade union or professional association representatives (1 nursing, 1 PT), and 14 managerial and frontline workers (7 nursing, 7 OT/PT). Two group interviews were conducted with nurses because of time constraints of participants, and the rest were individual interviews. The educators were recruited from two tertiary institutions and the front-line workers worked in diverse settings, including public hospitals (one nurse, one OT, two PT), a clinic (one nurse), psychiatry (five nurses, three OTs), and a community centre (one PT). Board members and officers in charge of professional registration and regulatory bodies declined interview invitations in this study.

All participants were informed on the information and risk of the study. Written consent was obtained from them. The protocol for the research project was approved by the Human Subjects Ethics Sub-Committee of The Hong Kong Polytechnic University (HSEARS20180111001).

### Contents of the key informant interviews

Based on the analytical framework mentioned earlier, the interviews focused on three major areas. The first area was about the professionals’ understanding of the meaning of cultural competence and challenges in their professional practices and current practices. The second area was concerned with professionals’ willingness and readiness to receive training regarding cultural competence. The third area focused on professionals’ viewpoints regarding potential means to strengthen cultural competence and promote training. Though the interview questions were set, participants were encouraged to share their viewpoints and experiences with minimal limitations imposed by the interviewers. Follow-up questions were asked based on their answers to more deeply explore their opinions.

### Data analysis

Interviews were audio-recorded after obtaining informed consent from each informant. Memo-writing for coding was conducted to explore and examine emergent insights constantly between members of the research team until theoretical saturation was reached (Charmaz, 1995, 2006). The themes were identified using theoretical thematic analysis (Maguire and Delahunt, 2017). A constant comparative approach to data analysis was used, involving studying the data on a case-by-case basis and coding the data line by line, then analysing the data across participants (in comparison), and collapsing the initial codes into categories that represent themes that emerged across the interviews. These procedures persisted until the point of theoretical saturation, and we sought additional participants to corroborate or elaborate on the findings so that new insights could be obtained through further sampling (Charmaz, 1995; Mills et al., 2006).

Scientific rigour and trustworthiness of data analysis were assessed by the principles of credibility, transferability, dependability, and confirmability as suggested by qualitative research experts (Lincoln and Guba, 1985; Sandelowski, 1986; Thomas and Magilvy, 2011). Member checking was used to strive for credibility. During the interviews, participants were asked to verify the interpretations emerged from interviews previously completed. To ensure transferability, this study provided thick descriptions on each theme by showing quotes supporting these themes. Dependability requires the research process to be traceable and clearly documented (Nowell et al., 2017). Therefore, research team members mutually audited the data collection and analysis process. For example, the transcripts of interviews conducted by one interviewer were objectively reviewed by another team members. The same process was adopted in the process of identifying codes and themes. To maintain confirmability of the results, audit trails were used. The team documented the transcript data and coding notes. Reflexive comments and relevant coding notes were shared and discussed among the team members till consensus on finalizing the key themes was reached.

The above process included examining obtained data sources to determine the congruency of findings, adopting a theoretical sampling approach which ensured that the age, education, and sociocultural backgrounds of the informants were diversified, reviewing justifications and interpretations of the findings by different research team members, and inviting independent researchers to respond to the findings by examining if they fit and confirm what had been found in the existing literature.

## Results

### Understanding of cultural competence

Key informants from the three health service professions described limited cultural competence when dealing with non-Chinese patients and limited cultural competence training. Understandings and practices of cultural competence differed across and within professions.

Most general nurses believed their levels of cultural competence were sufficient to address the basic needs of ethnoculturally diverse patients, but some admitted that they were not culturally sensitive enough in many circumstances.

‘*We are really not so clear about their cultural taboos and worried about making mistakes that we even [aren’t] aware of ourselves*’ (Nurse No. 2).

According to the impressions of our interviewees, most psychiatric nurses appeared to be more ‘human-centred’ compared with their general practice counterparts, as they spent more time communicating with patients and communicate more frequently with patients from diverse cultural backgrounds. Most local OTs and PTs, similarly as reported by our interviewees, were having considerable levels of understanding of ethnoculturally diverse patients, referring to family structures, common cultural taboos, and expectations for employment. However, they often lack knowledge of differences between ethnic groups and that of the implications and background of cultural norms.

‘*The general taboos, such as no pork for the Muslims, are well known to us, so we are able to avoid those mistakes. But for the details and implications of those differences, like why they wear headscarves… we really don’t know much*.’ (OT No. 7)

As with nurses, PTs seldom have close interactions with non-Chinese patients. They are generally less culturally sensitive in their routine working environment. Therapists working in community health centres generally spend more time with individual patients, including non-Chinese patients, so may be more culturally sensitive. And as OTs carry out training activities for patients and develop rehabilitation plans, they are relatively more aware of the needs of ethnoculturally diverse patients and are more skilful in cross-cultural practice based on their needs and unique situations of the non-Chinese clients. As for the nurses working in psychiatric settings, as they also need to work intensively with their patients to carry out rehabilitation activities and spend longer time in supporting the ethnoculturally diverse patients than nurses from other settings, they have also developed better understandings about the needs of these patients. One psychiatric nurse told the research team that psychiatric nurses and ‘case nurses’ spend time communicating with patients and are more sensitive to patients’ emotions. As they communicate more frequently with patients from diverse cultural backgrounds, they understand them better. The case nurse and other nurses and health professionals work closely with each other to handle each case:

‘*There is always a case nurse to take charge of each case. For example, when we intake an ethnic minority patient, he or she would be assigned to a case nurse. The patient would spend most of the time interacting with the case nurse, so we would check with the case nurse if we need to understand more about the patient. As we tend to have a closer relationship with our patients and communicate with them more frequently, gradually we can understand more about what they say*.’ (Nurse No. 3)

Professionals’ understandings of cultural competence vary according to their length of experience in the profession. The nurses and therapists mentioned in our interviews that ethnoculturally diverse clients are usually treated by senior practitioners as they are more experienced and confident. During informant interviews, more experienced professionals provided further elaboration on clients’ cultural characteristics and uniqueness, and challenges to serving culturally diverse groups.

Nurses and PTs reported that during university studies they received some training on general ethical issues related to respect for patients and equality, and common cultural taboos were mentioned. However, specific skills and attitudes for implementing professional values were not thoroughly explored. OTs also reported no specific training on cultural competence or cultural sensitivity in local tertiary institutions. On-the-job trainings were also limited across the three professions.

Professionals also rely on interpersonal knowledge transfer with colleagues. For example, nurses might consult those who spend more time with diverse patients in order to gain cultural understanding. PTs and OTs also referred to discussions with colleagues or other medical professionals about personal experiences in providing services for non-Chinese patients

‘*There are no specific programmes to equip the colleagues [on cultural competence], we rely on the existing guidelines most of the time. Our norm is we would transfer our experiences and skills to the student therapists and our juniors*.’ (OT No. 5)

The lack of in-depth training on cultural competence and reliance on (inter)personal experience can transmit inaccurate information and contribute to bias and stereotyping against non-Chinese patients. One therapist mentioned the difference between ‘first person’ and ‘second hand’ experiences in cultural exchanges.

‘*We very much rely on the experiences of other colleagues. But there could be distortions and biases. If we encounter more [non-Chinese patients], there could be less stereotyping and different ways of handling things*.’ (PT, No. 9)

### Needs and challenges in providing culturally competent services

Nurses and therapists reported that interpretation services in public hospital were rarely available to them and that they often relied on body gestures and translation by patients’ family members. PTs reported that they often needed to spend more time explaining different types of treatment through body language and pictures. Translation services in the Hong Kong medical sector are mainly delegated for use by medical doctors, and some therapists only seek to access interpretation services on a ‘tag along’ basis when an interpreter has received a service order from a medical professional.

Differences in communication challenges exist *within* professions. Some general nurses rarely encounter challenges in communicating with non-Chinese patients because they can rely on body language or ask patients’ family members to translate for them. However, psychiatric nurses have relatively closer relationships with non-Chinese patients and want to build up more cohesive rapports with patients and families, and communication barriers due to language differences are a concern.

‘*I once had an Indian patient who spoke neither Chinese nor English… The daughter translated from Urdu to English word by word for us, so we relied on this mode of communication to understand the patient’s basic information. But when that patient tried to say something and she could not write anything on paper, my colleagues felt annoyed as they did not understand what she tried to express*.’ (Nurse No. 3)

OTs explained that in many circumstances, they needed to make more effort to explain Hong Kong work cultures to non-Chinese patients and to guide them to adapt to the wider social context.

‘*For example, we always emphasize the importance of punctuality, but some of the ethnic minorities may not understand why it is so important. In this regard, we need to understand different issues from their perspectives*.’ (OT No. 8)

For nurses, rigid and stressful everyday working environments in hospitals also restrict culturally competent care. Rigid official standardized protocols do not encourage patient-centred practice in frontline contexts, let alone the care of non-Chinese patients’ feelings and needs. Finally, busy environments limit tailored care.

‘*It is quite challenging to be flexible in actual practice and get used to the norms of different people… There are already so many patients sleeping in the hospital corridors, how could they have extra time and energy to be culturally competent?*’ (Nursing Educator, No. 13)

Workforce shortages present another grave problem for the nursing sector. For a number of years, the number of nurses in Hong Kong hospitals has been insufficient, so it is difficult for hospitals to implement a patient-centred approach similar to that used in some Western countries.

‘*I think [the level of cultural competence] is more related to the manpower of nurses in Hong Kong. Very different from other countries where the nurse-patient ratio might be as low as 1:4, but it could be 1:12 here in Hong Kong*.’ (Nursing educator No. 12)

Finally, a lack of cultural competence and a failure to accommodate needs of diverse patients at the management level further hinder nurses’ abilities to provide culturally competent services.

‘*In the public hospitals, when a non-Chinese patient asks for Halal food, the hospital could only provide a vegetarian meal. I have reflected such situations to the hospitals. They replied that they do provide Halal food, but it depends on the supply. When the supply is here, they can provide it. When it isn’t, there is nothing they can do. Such situation also reflects how much the hospitals value [cultural competence]*.’ (Nurse No. 1)

### Willingness to receive training on cultural competence

Healthcare professionals’ willingness to receive further training on cultural competence varies from profession to profession. Compared to most nurses and PTs, OTs generally had a greater willingness to receive further training on cultural competence, as it appears that the nature of their work requires them to understand clients’ background, mindsets, and cultural norms and to help them to adapt to local social and learning environments. In general, professionals who need to serve more ethnoculturally diverse clients and those with more diversified roles and service settings are more willing to receive training on cultural competence. A therapists reflected on the need of receiving training:

‘*We would like to understand more about their medical history, like whether they would trust the doctors more than us, or they are afraid of acupuncture. These would enable us to arrange their care plans properly and introduce them some therapies that are more common locally*.’ (PT No. 9)

Most nurses (both trainers and frontline nurses) and PTs felt that for most professionals, cultural competence was generally not a priority so they did not find an imminent need for training. PTs reported that they normally did not encounter many difficulties when serving ethnoculturally diverse patients in their usual work settings. Most therapists believed that it was enough for them to fulfil their duties by providing necessary treatments for all clients equally. However, nurses explained that they did not oppose receiving such training and agreed that there should be more elements related to cultural competence included in future curricula. All OT professionals explained that they were willing to receive training on cultural competence, but they suggested that in order to encourage more colleagues to enrol, programmes should be specially designed and provided for OTs rather than inviting them to join training activities for all medical professionals.

### Facilitators and barriers to cultural competence training

Nursing professionals noted that subsidies for enrolling in training activities would be a major facilitator because they might need to take leave or spend off-duty hours to attend the training. Nursing and therapy professionals suggested that practitioners would also be encouraged to receive training if they received incentives such as accreditations, certificates, and other forms of professional recognition upon completion of training. As a nursing professional explained, it would be helpful to highlight the potential benefits of completing training, such as obtaining credits and accreditations:

‘*We need to keep score for professional development. If we study those courses, we may get extra score for it. It is not compulsory. But for those who want a promotion, they will try to get a higher score*.’ (Nurse No. 1)

It was suggested that training programmes should be designed for specific professions and the content should not be too general. For example, PTs and OTs should not be asked to attend workshops and programmes for general medical service staff. Most PTs and OTs also suggested that training activities should focus more on practical issues than theoretical concepts. For example, as one therapist suggested,

‘*It would be very interesting if the course talks about how to handle complaints from non-Chinese patients because we lack the ability to handle them and handling complaints is the most time-consuming duty. They would be very interested if the course may facilitate them to handle complaints more effectively*.’ (PT No. 9)

Common barriers to receiving training identified by nursing and therapy professionals included tight working schedules and stressful working environments. This illustrates the significance of institutional or system-wide factors, but not only individual attitudes, in influencing professionals’ engagement in cultural competence training. Additionally, some professionals might not be interested in enrolling in training if they did not regularly encounter and serve non-Chinese clients as they might not consider cultural competence training a priority or see the need to enhance their knowledge and skills.

### Effective means of promoting cultural competence

Key informants from all the professions recommended that training on cultural competence should start early in programmes offered by tertiary institutions, rather than introducing intensive training programmes after practitioners’ entry into the job market. Both nurses and PTs explained that they were busy at work and might not have time to join training courses and activities related to cultural competence, so it would be more feasible to implement training for students in tertiary institutions.

When discussing training for students, key informants from all the three professions suggested that content related to cultural diversity in Hong Kong and professional practice in diversified cultural settings should be integral parts of training curricula and subsumed into existing courses and practicums, rather than establishing new specialized courses (either compulsory or elective).

‘[A specialized course] *may not need to take a whole semester, as many students would see this as a small topic and not attractive enough. It should be enough for us to spend one to two sessions. Most importantly, we need to let them develop sensitivity and awareness, so that we could talk about enhancement of cultural knowledge later*.’ (OT No. 8)

Nursing professionals suggested that instructors should mention potential challenges in working with ethnoculturally diverse patients in medical service settings, and that front-line nurses with cultural experiences could be invited to share their experiences with students. As a nursing educator pointed out, cultural competence training programmes should combine knowledge and practice. Students can learn basic cultural knowledge in seminars and attend service learning and/or exchange programmes to practise what they have learned. Similarly, PT/OT educators and frontline therapists suggested that there should be more interactions between students and ethnoculturally diverse clients through specific placement settings, volunteering experiences, and site visits, although such opportunities are currently limited.

Key informants also discussed best practices for ongoing professional training. For example, nursing professionals suggested that workshops could be offered to frontline nurses in order to enhance their awareness of cultural diversity and nurture their cultural competence. As with training in tertiary institutions, former non-Chinese patients and experienced nurses could be invited to share practical issues related to cultural taboos and case examples. Additionally, one key informant suggested that instructors should guide nurses to reflect on their practices in workshops, as self-reflection is considered an essential step to enhance awareness of racism and consequently minimize racist attitudes and behaviours.

Besides, a number of OT/PT professionals suggested that it would be helpful to provide ongoing training through online courses (involving video clips, quizzes, and reading materials) as this mode of learning would allow more flexibility. As another professional explained, their heavy workload might not allow professionals to travel to other places to attend training and therefore online training would be more suitable. Another therapist noted that:

‘Training activities should not be too long, as the number of ethnic minority clients is relatively low… [and] some colleagues might not have time to attend long course.’ (OT No. 5)

Additionally, online courses could make it easier to retrieve materials about cultural competence required at different points in time.

## Discussion and recommendations

This study has three major objectives, namely to examine understandings and implementation of cultural competence among healthcare professionals, identify facilitators and barriers related to these professionals’ readiness to receive training, and examine how cultural competence could be more effectively promoted and enhanced among the healthcare professionals. In terms of understanding and implementation of cultural competence, the results show that there are generally much room for improvement in the training of cultural competence for all the three healthcare professions. Healthcare professionals encounter numerous challenges of in-depth cultural knowledge and skills in working with ethnoculturally diverse groups, high reliance on standardized protocols without cultural considerations and interpersonal exchange, language barriers, and insufficient resources to support culturally competent service. Nurses and PTs generally feel that it is sufficient to fulfil their duties by following standardized procedures and protocols for patients from all cultural backgrounds, whereas OTs often need to engage in more personal communication with patients. The extent of the implementation of culturally competent practice varies across service settings. For example, psychiatric nurses might consider themselves more culturally sensitive than general nurses, because they need to build up trust and maintain smooth communication with patients and their families to facilitate rehabilitation.

Regarding facilitators and barriers related to the healthcare professionals’ readiness to receive training on cultural competence, our results found that general nurses and PTs generally have a lower willingness to receive such training, as they deem themselves competent in working with non-Chinese patients. The enhancement of cultural competence is not considered a priority among these healthcare professionals’ busy work schedule. Together with the point about the inconsistent provision of Halal food to patients, these are typical examples of systemic barriers to culturally competent services. In addition, general nurses and PTs often face language barriers and various forms of cultural shocks when encountering the cultural norms of ethnoculturally diverse clients, such as taboos in physical contacts with patients, gender relations, food preferences, and patients’ proficiency in English or Chinese languages. They either feel helpless or over-conscious about cultural taboos due to lack of previous skills training and knowledge about these patients. As such, they often choose not to interact closely with these patients while fulfilling their duties. OTs seem to have a higher willingness since they need to work closely with clients during job seeking and rehabilitation processes. Professionals’ willingness would be stronger if they encounter more non-Chinese clients in their service settings and if their daily tasks require closer working relationships with the ethnoculturally diverse clients.

When it comes to effective means of promoting training and practices related to cultural competence in healthcare settings, nursing and therapy professionals commonly suggested that training content should be tailored to specific professions and that training subsidies, flexible working hours, and incentives for training should be offered. They also suggested to provide more assistive materials about culturally competent practices. Nurses would prefer attending one-off practical workshops, whereas PTs and OTs preferred receiving online training due to their tight working schedules. These suggestions point to the significance of wider institutional or systemic factors in affecting healthcare professionals’ willingness and ability to receive cultural competence training.

A number of recommendations have emerged from the findings to strengthen cultural competence training for healthcare professionals. The first calls for the promotion of an infusion model of cultural competence training. Professional training on cultural competence should begin early in tertiary institutions where future professionals receive training. Similar approaches are discussed in the literature on overseas immersion approaches for therapists (Ekelman et al., 2003) and nursing students (Snyder et al., 2008; Chen et al., 2013). Echoing the Cultural Competence Attainment Model (McPhatter, 1997), the findings suggest that it is important to implement a focused, systematic, and reflective process of ongoing development of skills and worldviews. Therefore, in Hong Kong, professional education curricula should adopt an ‘infusion model’ that incorporates cultural competence concepts and knowledge (including cultural sensitivity, knowledge of the needs of ethnoculturally diverse clients, and concepts of diversity and equality) into core and mandatory courses taken by future professionals. This would ensure that all future healthcare professionals have a fundamental level of competence for working with ethnoculturally diverse groups. Senior workers experienced in working in culturally diverse settings could share practical frontline experiences, and individuals from ethnocultural communities should be invited to share first-hand experiences of accessing health services and interacting with professionals. Through these exchanges, both current and future healthcare professionals can benefit from first-hand experiences of interacting with clients with different backgrounds, thereby attaining, what the Cultural Competence Attainment Model (McPhatter, 1997) suggests, grounded knowledge base and better cultural competence.

The second recommendation concerns the enhancement of on-the-job training for healthcare professionals. The importance of such training echoes the emphasis of the Cultural Competence Attainment Model on cumulative skill proficiency (McPhatter, 1997). Such training benefits not only practitioners themselves but also the service quality of agencies. Employers should encourage practitioners to enrol and participate in training programmes during work hours. In addition, incentives should be available for practitioners who enrol in training programmes (such as subsidies and accreditations to recognize training participation and achievements) and training should be counted in point systems associated with longer-term promotion and employment.

The third recommendation calls for systemic and institutional changes in all healthcare professions to remove the systemic barriers. As mainstream healthcare systems, services, and settings are designed mainly to cater to the needs of local Chinese populations, they overlook the quality of care and social integration for non-Chinese clients. To address language barriers, public notices and leaflets about health services should all be available in English and Chinese, and for services with a substantial percentage of ethnoculturally diverse clients and all printed materials should be available in other languages. Most importantly, patient voices from the culturally diverse communities should be systemically included through the establishment of a patient consultation mechanism so that their needs and preferences are better reflected and communicated with the service providers. For professional and regulatory bodies, existing codes of practice and protocols for all the three professions include only vague provisions, stating that professionals should provide services equally to each user regardless of ethnic and cultural background. They are, however, not sufficiently proactive in promoting culturally competent practice. Emphasizing the value of cultural competence in the codes of practice and professional protocols can, as suggested by the Cultural Competence Attainment Model (McPhatter, 1997), enlighten local healthcare professionals’ consciousness of respecting clients with different backgrounds and attending to cultural differences.

The fourth recommendation calls for the establishment of a culturally competence lens for policy making and program delivery. The existing Race Discrimination Ordinance could be strengthened to ensure the accessibility and rights of clients in using health services. Government and public organizations’ efforts and resource investment to ensure equity and accessibility for culturally diverse communities in service use should also be monitored and reported on regular basis. Frameworks adopted in other countries (e.g. United Nations Educational, Scientific and Cultural Organization, 2011; Hispanic Children and Families, 2017) highlight some key features for analysing and developing policies and programmes, including examining the legislative and political environment and socio-economic data on ethnoculturally diverse clients and eliminating barriers that limit the access of ethnoculturally diverse clients through enhanced sensitivity to cultural and linguistic differences. These are useful to policy makers and service providers, who need to review human resource deployment and service delivery.

The final recommendation concerns wider social changes via community education. Ethnoculturally diverse groups are not always portrayed positively in mainstream media and public discourses in societies (Ahmed and Matthes, 2017), including in Hong Kong (Sung, 2005; Erni and Leung, 2014; Jackson and Nesterova, 2017). Prevailing social mindsets and perceptions about ethnoculturally diverse groups, including stereotypes of and bias towards ‘ethnic minorities’, may influence healthcare professionals’ ideas about cultural competence training. The government, the public sector, and the media should help to facilitate an inclusive social environment and promote equal opportunities and the elimination of racial stereotyping. This might be accomplished through public education programmes focusing on promotion of inclusion and diversity and provision of opportunities for Hong Kong residents of different ethnocultural backgrounds to interact and challenge stereotypes.

## Study strengths and limitations

This study explored views from different sectors, thereby providing a comprehensive understanding of receptivity and readiness for cultural competence training among healthcare professionals. Also, the diversity of the samples has enabled us to identify the most suitable way for implementing cultural competence training for healthcare professionals. The findings produce practical implications for developing training for healthcare professionals in other countries. However, the study failed to further explore the different needs of cultural competence training among healthcare professionals within specific disciplines (e.g. psychiatric nurses versus general nurses) because they have different work contents, which could be explored more in future studies. Also, the number of participants from different sectors and disciplines was not even, which may influence the results. This should be addressed in future studies.
